# Older spiny mice (*Acomys cahirinus*) have delayed and spatially heterogenous ear wound regeneration

**DOI:** 10.1242/bio.060565

**Published:** 2024-10-10

**Authors:** Justin A. Varholick, Jazmine Thermolice, Gizelle Godinez, Vanessa Dos Santos, Rishi Kondapaneni, Malcolm Maden

**Affiliations:** ^1^ Department of Biology, University of Florida, Gainesville, FL, USA; ^2^ Department of Molecular Genetics and Microbiology, University of Florida, Gainesville, FL, USA; ^3^ McKnight Brain Institute, College of Medicine, University of Florida, Gainesville, FL, USA; ^4^ Department of Psychology, University of Florida, Gainesville, FL, USA; ^5^ Genetics Institute, University of Florida, Gainesville, FL, USA

**Keywords:** Regeneration, Aging, Spiny mouse, Ear, Nerve degeneration

## Abstract

The African spiny mouse (*Acomys cahirinus*) is a unique mammalian model of tissue regeneration, regenerating 4 mm ear-hole punches with cartilage, adipocytes, hair follicles, and muscle. However, the time to regenerate ear tissue varies from 20 to 90 days and muscle regeneration is inconsistent. Some report that older spiny mice have delayed regeneration without investigation on the regenerative capacity of muscle. We thought that delayed regeneration and inconsistent muscle regeneration could be linked via age-related nerve degeneration. While the current study found that spiny mice aged 6–9 months had delayed regeneration compared to 3–4 month-old spiny mice, the capacity of muscle regeneration was unrelated to age, and there was little evidence for age-related nerve degeneration. Instead, the regeneration of muscle, cartilage and adipocytes was spatially heterogeneous, declining in amount from the proximal to distal region of the regenerated tissue. Also, cartilage regeneration in the distal region was decreased in ≥22-month-old *Acomys* and adipocyte regeneration was decreased in those older than 6 months, compared to 3–4 month olds. While the underlying mechanisms for delayed and spatially heterogenous regeneration remain unclear, age and the spatial region of the regenerated tissue should be considered in experimental designs with spiny mice.

## Teaser

**Summary:** Older Spiny mice took longer to regenerate ear wounds with less cartilage and fewer adipocytes. All regenerated tissue had less cartilage and muscle than uninjured tissue, declining proximal to distal.

## INTRODUCTION

African spiny mice (*Acomys spp.*) are the first known mammals capable of scar-free healing and regeneration of multiple tissues and organs. An initial study in 2012 discovered scar-free healing and regeneration of the skin and the ear-pinna (Seifert et al., 2012). This finding was of major significance since mammals were previously thought to be incapable of regenerative healing beyond embryonic development or their digit-tips (Goss, 1980). Subsequent studies over the past decade have further demonstrated that *Acomys* regenerate skin after burn wounding (Maden, 2018), muscle after repeated cardiotoxin injuries (Maden et al., 2018), the spinal cord after hemi-crush or transection (Nogueira-Rodrigues et al., 2022; Streeter et al., 2020), and the kidney after ischemia (Okamura et al., 2021). They also have remarkable resistance to myocardial infarction (Koopmans et al., 2021; Peng et al., 2021; Qi et al., 2021). This makes them an important model organism for understanding tissue regeneration in mammals (Seifert and Maden, 2014).

While the regenerative capacity of *Acomys* is remarkable, studies on the ear-pinna have reported inconsistent timing and quality of regeneration. Specifically, the time to regenerate – or close – a 4 mm ear-hole punch ranges from 20 to 90 days within and between studies (Gawriluk et al., 2016; Matias Santos et al., 2016; Seifert et al., 2012; Simkin et al., 2017) (see Table 1). This variability could bias experimental results and contribute to irreproducibility if neglected from the experimental design. For example, previous studies measuring molecular pathways underlying regeneration in *Acomys* have extracted RNA along multiple time points after injury (e.g. 5, 10, 15, and 20 days post injury) (Gawriluk et al., 2016). If the timing of regeneration widely varies between animals, then animals within a time point (e.g. 10 days post injury) may be at different phases of regeneration, and animals between time points (e.g. 5 and 10 days post injury) may be at more similar phases of regeneration, albeit this remains to be shown. In addition, there is variability in the quality of regeneration between studies. The original research reported no muscle regeneration in the ear (Seifert et al., 2012). Meanwhile, subsequent studies and published figures show muscle in the regenerated zone (Matias Santos et al., 2016; Seifert and Muneoka, 2018). There are also significantly elevated levels of embryonic myosin (i.e. *Myh3*) and other muscle-related mRNA expression during regeneration, suggesting that ear muscle regenerates (Gawriluk et al., 2016; van Beijnum et al., 2023). Inconsistent muscle regeneration would also contribute to irreproducibility in molecular experiments, for example. To better understand this variability in timing and regeneration, the factors contributing to delayed regeneration and differences in muscle regeneration must be uncovered. Once these factors are understood, they can be included in experimental designs and statistical plans to understand the underpinnings of ear tissue regeneration in *Acomys*.

**Table 1.**
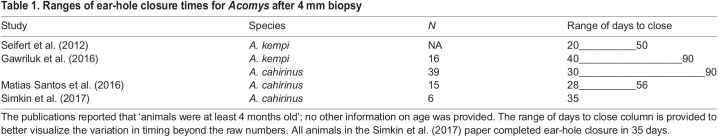
Ranges of ear-hole closure times for *Acomys* after 4 mm biopsy

Various factors likely affect the timing of *Acomys* ear-pinna regeneration, but the most salient factor currently is age. Some supplemental suggested that blood draws or lactation can speed up regeneration in females (Gawriluk et al., 2016), but only one of four studies included blood measurements or lactating females. Two studies suggested that age is a significant factor because older *Acomys* had delayed regeneration compared to younger *Acomys* (Brewer et al., 2021; Oyake and Mekada, 2024). Unfortunately, the injuries from these studies on age were limited to 2 mm biopsies. The 2 mm biopsies are incomparable to 4 mm biopsies because lab mice (*Mus musculus*) can heal 2 mm injuries via closing but not 4 mm biopsies (Rajnoch et al., 2003). Interestingly, most studies (see Table 1) have neglected to consider whether age is a variable in their experiment, only reporting that animals were greater than 4 or 4.5 months old. In laboratory experiments, this neglect of age may be due to the lengthy gestation and small litter size of *Acomys* (i.e. 40-day gestation with 1–4 pups per litter). Without a large breeding colony, lengthy gestation time and small litter sizes, can make it difficult to have enough animals to run an experimental batch. This encourages some scientists to run experimental batches of animals with multiple ages. Research on the effect of age following the common 4 mm biopsy punch is therefore necessary to determine whether age is an important factor to include in experimental designs and statistical analyses when studying *Acomys* ear-pinna regeneration.

**Fig. 1.**
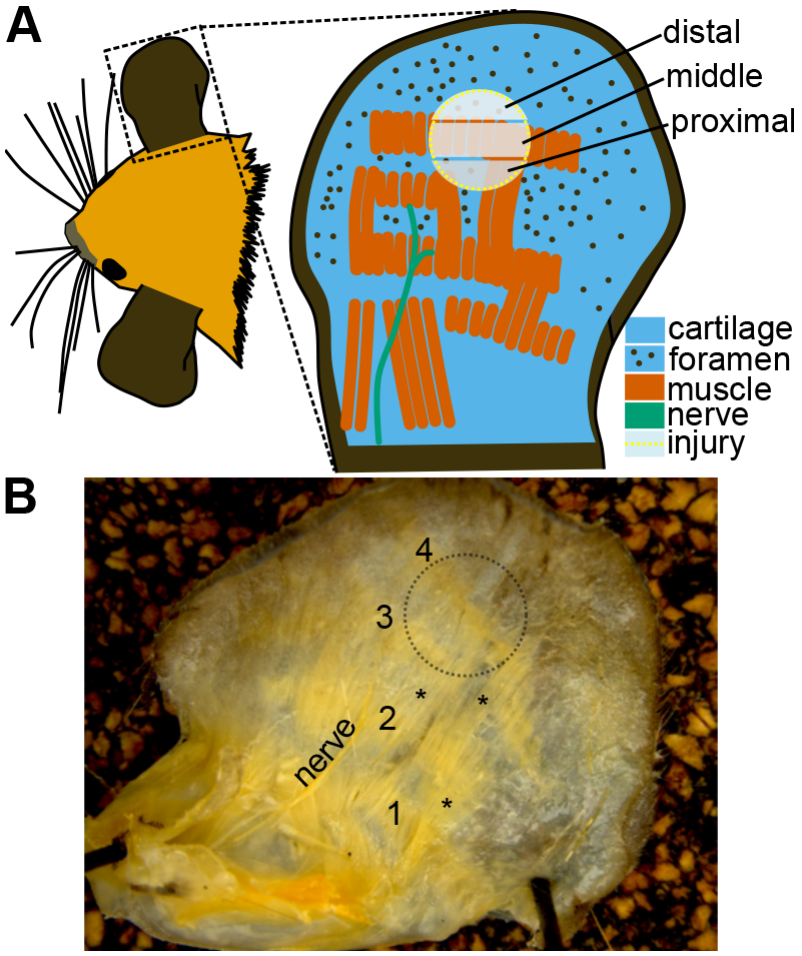
**Representation of ear-pinna anatomy.** (A) Diagram showing the general anatomy of the cartilage, foramen, muscle, and major nerve (branch of the auriculotemporal nerve) relative to the injury (outlined in dotted yellow). The injury zone is then divided into three zones: proximal, middle, and distal. The muscles and nerves were determined from Fig. 1B, while the cartilage and foramen were determined from previous studies (Allen et al., 2023preprint). (B) Photograph referenced for the musculature of the ear-pinna. The muscles are in four horizontal bands (labeled 1–4). These bands are sometimes interconnected by muscle (*). Notice the lack of muscle in the distal zone on the biopsy injury outline. The ear was fixed in 10% neutral buffered formalin, and the dorsal skin was gently peeled off with a fine pair of tweezers.

One reason there could be wide variability in muscle regeneration in the ear is the wide spatial variability in tissue types across the ear, proximal to distal. For example, there are more muscles and less cartilage foramen (i.e. openings in the cartilage) in the proximal region of the ear, closest to the head, than the distal region (Fig. 1A,B) (Allen et al., 2023preprint; Heuser and Tenkova, 2020). Since there is little to no muscle in the distal region of the ear prior to wounding, inconsistent findings on muscle regeneration may depend on the spatial region of the tissue analyzed. That is, some studies may do histology in the proximal region of the regenerated tissue while others analyze histology on the distal region. A recent study measuring gene expression of the proximal, middle, and distal regions during regeneration determined that the proximal region had more upregulation of genes associated with cell proliferation, muscle, and adipocytes than the distal region (van Beijnum et al., 2023). Also, there was less gene expression with chondrocytes and sebaceous glands in the middle region. Thus, muscles, for example, may only regenerate in the proximal region. Unfortunately, this study (van Beijnum et al., 2023) lacked histological or proteomics data on the tissue after ear hole closure (i.e. regeneration) was complete to determine whether muscles or other tissues were regenerated.

Another possible explanation for variability in muscle regeneration could be partial nerve damage from age-related nerve degeneration. Studies in axolotls have partially damaged nerves and found delayed regeneration coinciding with missing digits, reduced cell proliferation, and elevated levels of cell death (Egar et al., 1982; Maden, 1981; Wells et al., 2021). Peripheral nerves of mammals are known to partially degenerate or become damaged with age, commonly referred to as peripheral neuropathy (Bolton et al., 1966; Cauna, 1964; Gardner, 1940). This age-related partial nerve damage delays wound healing in humans (Clayton and Elasy, 2009) and would likely delay tissue regeneration (Yun, 2015). While some studies suggest that *Acomys* have attenuated signs of aging (Bellofiore et al., 2021; Wong et al., 2020) and can live up to 6 years (Morrison et al., 1977), very little is known about their aging (Bellofiore et al., 2021; Dawirs et al., 1992; Haughton et al., 2016; Wong et al., 2020). Thus, it is possible that delayed regeneration in older *Acomys* is related to inconsistent reports of muscle regeneration due to age-related partial nerve damage in the older animals. Indeed, nerves are required for ear regeneration in *Acomys* (van Beijnum et al., 2023), similar to limb regeneration in axolotls (Kumar and Brockes, 2012).

Here, we investigated the relationship between age, timing, and regeneration quality in the *Acomys* ear*.* We also investigated the presence of age-related partial nerve damage in the auriculotemporal nerve innervating the *Acomys* ear. We measured the time to complete regeneration (i.e. closure of a 4 mm biopsy punch to the ear-pinna) in *Acomys* ranging in age from 3 to 34 months. After regeneration, the tissue was fixed and serially sectioned to determine the quality of the regenerated cartilage, adipocytes, muscle fibers, and hairs across the proximal to distal regions compared to uninjured tissue. In a separate study, the auriculotemporal nerves were dissected from 6 and ≥22 month-old *Acomys,* and semithin (0.5 µM) sections were stained with Toluidine Blue for signs of age-related nerve damage.

## RESULTS

### Age is related to delayed timing of ear-hole closure

**Fig. 2.**
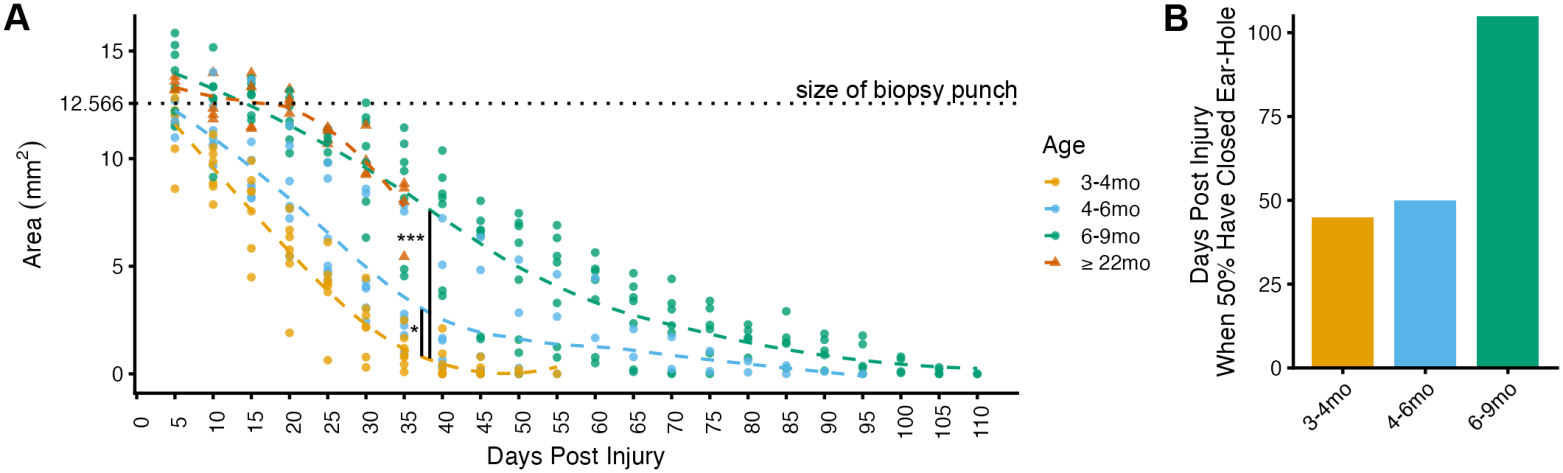
**Time to close ear-hole for multiple ages.** (A) Scatterplot of the decreasing ear-hole area across days post injury according to age of the animal. Each point per day represents one animal. A polynomial line per age category shows the trend of the data. A linear mixed-effects model on animals between 3 and 9 months old (*n*=21, seven per age group) at the time of injury indicated that age was related to time to regenerate [*F*(2, 20)=19.584, ***P<0.001], with 4–6-month-old and 6–9-month-old animals taking longer to regenerate than 3–4 month olds (t=2.500, *P=0.020; t=6.145, **P<0.001). Animals ≥22 months old (triangles, *n*=4) were excluded from the statistical analysis, due to attrition. (B) Bar chart showing the days post injury when 50% of animals in an age group have closed the ear-hole. Animals ≥22 months old were not included since we stopped tracking their ear-hole closure after 35 days.

After a 4 mm biopsy punch to the ear-pinna, the total size of the ear-hole was measured every 5 days until the hole closed (*n*=21, seven animals per age group; 3–4 months, 4–6 months, and 6–9 months). The area of the hole for each day, for each animal, was analyzed using a linear mixed-effects model with age as an ordinal fixed effect and days as a random effect. This analysis determined that age had a significant effect on regeneration [*F*(2, 20)=19.584, *P*<0.001] (Fig. 2A). Post-hoc comparisons of the age categories showed that both 4–6-month-old (t=2.500, *P*=0.020) and 6–9 month-old (t=6.145, *P*<0.001) animals took longer to regenerate than 3–4 month-old animals. This effect appears limited to 6–9-month-old animals, however, when comparing the time when 50% of the animals have a closed ear-hole in each group (Fig. 2B). An additional study on *Acomys* ≥22 months (*n*=4) determined that they followed a similar healing trajectory to the older 6–9-month-old animals (Fig. 2A, labeled as triangles). Unfortunately, this data was limited to days 0 to 35 days post-injury due to the COVID-19 shutdown. The animals ≥22 months old were not included in the linear mixed-effects model analysis due to lack of data.

### *Acomys* regeneration is spatially heterogeneous, with some differences in quality with age

After ear-hole closure was complete, a subset of ears (*n*=20, four per age group and four control uninjured ears) was histologically examined for differences in the quality of the regenerated tissue. Multiple sections were analyzed for each animal along the proximal to middle to distal regions of the regenerated tissue. To compare the uninjured and injured tissue, a region of interest (ROI) was compared. The ROI for injured animals was the regenerated tissue, while the ROI for uninjured tissue was a predefined 4 mm zone in the same area as the ROI for the injured animals.

### Cartilage percentage and counts

**Fig. 3.**
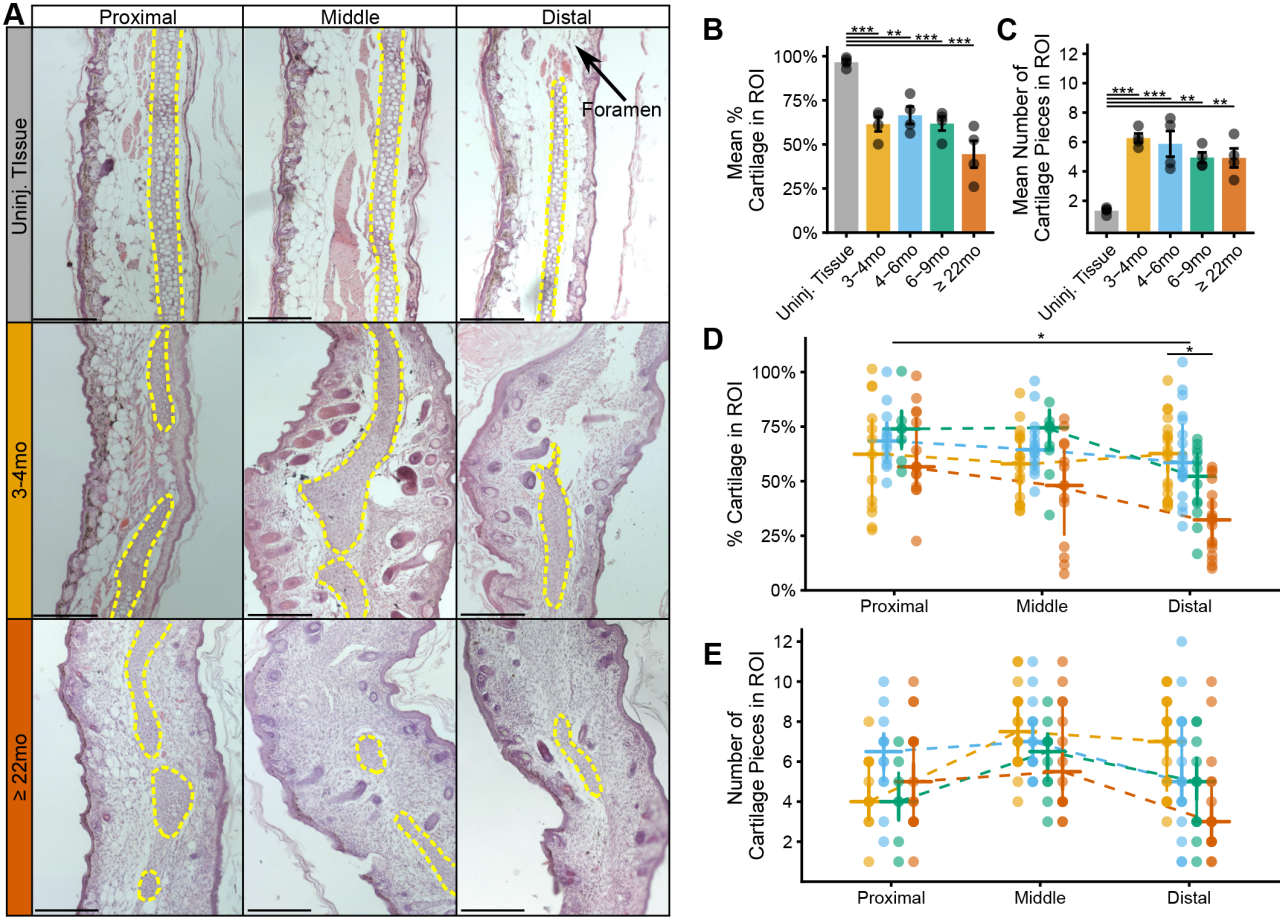
**Quality of cartilage percent and counts.** (A) Panel of histological images of uninjured tissue (*n*=4 animals) and regenerated tissues of 3–4-month-olds and ≥22-month-olds across proximal, middle, and distal regions (*n*=4 animals per age group). The cartilage is outlined in yellow dotted lines, and one foramen in the uninjured tissue is marked with an arrow. Other ages are excluded from this figure for brevity. All images from left to right are from the same animal. Scale bars: 250 µM. (B) Bar chart of mean values with error bars of standard error of the mean, showing that all regenerated tissue had significantly less cartilage than the uninjured tissue, as determined by post-hoc Tukey tests from a one-way ANOVA. **P<0.01 and ***P<0.001, adjusted for multiple comparisons. (C) Bar chart of mean values with error bards of standard error of the mean, showing that all regenerated tissue had significantly more pieces of cartilage (i.e. more foramen or holes in the cartilage). (D) Line chart of median values with the first and third quartile for each age (colored according to 3B) across proximal, middle, to distal (x-axis). The precent of regenerated cartilage significantly decreased from proximal to middle to distal regions**, as determined by a linear mixed-effects model. In the distal region, ≥22-month-old animals regenerated less cartilage than 3–4-month-olds**, as determined by fixed effect comparisons using a Wald t-distribution. (E) Line chart of median values with the first and third quartile for each age (colored) across proximal, middle, to distal (x-axis). A linear mixed-effects model determined there were no significant differences in number of cartilage pieces across the spatial regions, and regardless of age. The analyses in this figure and Fig. 4 use samples from the same *n*=4 mice for each age category, and some of the images in A depict sections from the same samples as shown in Fig. 4A and C.

Differences in the percentage of cartilage and counts of cartilage pieces (i.e., an estimate of cartilage foramen) for each age category were compared to uninjured tissue (Fig. 3A). The percentage of cartilage was defined as the length of cartilage within a histological section divided by the size of the ROI. Counts of cartilage were made on the same ROIs. Post-hoc tests from a one-way ANOVA revealed that all regenerated tissue had significantly less cartilage than uninjured tissue: 3–4 months old (diff=−35.10%, *P*=0.001), 4–6 months old (diff=−30.04%, *P*=0.004), 6–9 months old (diff=−34.77%, *P*=0.001), and ≥22 months old (diff=−52.09%, *P*=0.001) (Fig. 3B). The cartilage was also broken into more pieces, or had more foramen, compared to uninjured tissue: 3–4 months old (diff=4.94, *P*=0.001), 4–6 months old (diff=4.55, *P*=0.001), 6–9 months old (diff=3.62, *P*=0.002), ≥22 months old (diff=3.59, *P*=0.002) (Fig. 3C).

**Fig. 4.**
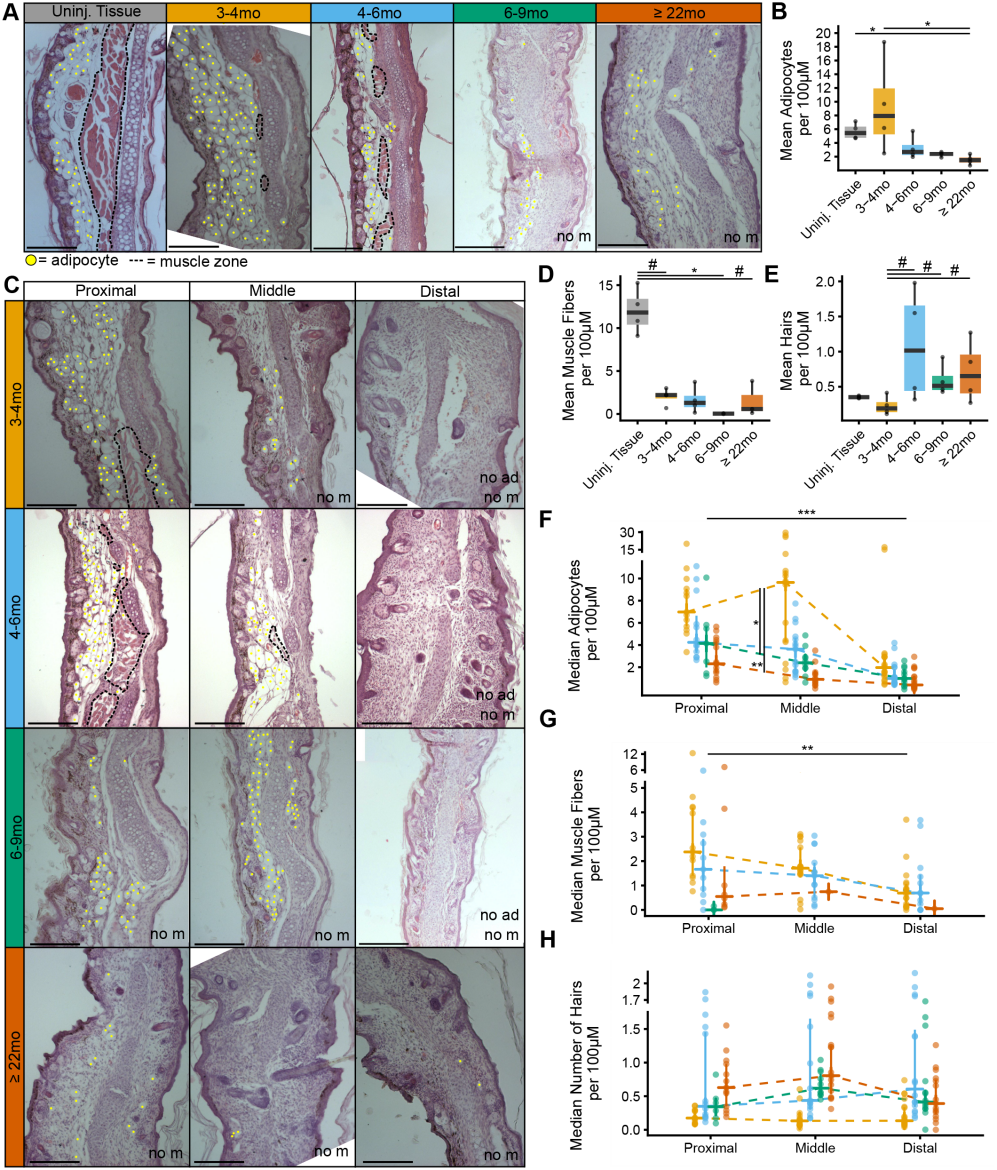
**Counts of adipocytes, muscle fibers, and hairs.** (A) A panel of histological images of uninjured tissue and regenerated tissues from each age category (3–4 months old, 4–6 months old, 6–9 months old, and ≥22 months old; *n*=4 animals for each age category). Adipocytes are denoted with yellow circles, and muscle is outlined in black. “no m” indicates there is no muscle in section. Scale bars: 250 µM. (B) Boxplot showing that uninjured tissue and 3–4 months old regenerated tissue has more adipocytes than regenerated ≥22 months old (Kruskal–Wallis test followed by a Dunn's test). *P<0.05, adjusted for multiple comparisons. (C) Panel of histological images of each age category across the proximal, middle, and distal regions. The annotations following 4A, plus “no ad” indicates there are no adipocytes. All images from left to right are from the same animal. (D) Boxplot showing that the regenerated tissues have less muscle fibers than the uninjured tissue (Kruskal–Wallis test followed by a Dunn's test). #*P*, uncorrected <0.05. (E) Boxplot showing that animals older than 3–4-months appear to have more regenerated hairs (Kruskal–Wallis test followed by a Dunn's test), albeit this was not significant after correction of multiple testing. (F) Line chart of each age (colored according to panels A-E) across proximal, middle to distal regions (x-axis). The percent of regenerated adipocytes significantly decreased across spatial position with more adipocytes in the proximal region in a linear mixed-effects model. Also, 6–9 month olds and ≥22 month-olds had fewer adipocytes than 3–4 months olds, as determined by fixed effect comparisons using a Wald t-distribution. ***P<0.001, **P<0.01. (G) Line chart showing that the number of regenerated muscle fibers decreased from proximal to middle to distal region, as determined by a linear mixed-effects model. (H) Line chart showing no significant changes in the number of regenerated hairs across spatial regions as determined by a linear mixed-effects model. The analyses in this figure and Fig. 3 use samples from the same *n*=4 mice for each age category, and some of the images in A and C depict sections from the same samples as shown in Fig. 3A.

Differences in the percentage of cartilage and counts of cartilage pieces were also compared across ages and proximal-middle-distal position. For these analyses, model comparisons were made across three linear mixed models with position nested within individuals as a random effect. The first model included position as a fixed effect, the second model added age as a fixed effect, and the third model added the interaction of position and age as a fixed effect. The best model for the percent of cartilage included position and age (i.e., the second model). Thus, there was an effect of position [*F*(1,17)=11.488, *P*=0.003] and age [*F*(3,12)=6.309, *P*=0.008], but no interaction between the two. Post-hoc analyses of the fixed effects indicated that the percent of regenerated cartilage declined from proximal to distal position (t=−3.389, *P*=0.003) and that ≥22 month olds had less cartilage than 3–4 month olds in the distal region (t=−3.406, *P*=0.006) (Fig. 3A and D). The best model for the pieces of cartilage was the first model, revealing no significant differences in pieces of cartilage in relation to position, age, or the interaction of age and proximal to distal position (Fig. 3E).

### Counts of adipocytes, muscle fibers, and hairs

The number of adipocytes, muscle fibers, and hairs per 100 µM were then compared for each tissue across ages and compared to uninjured tissue. This data was non-normally distributed, requiring a Kruskal–Wallis test followed by a pairwise Dunn's test. For adipocytes, the analyses indicated that those ≥22 months old had significantly fewer adipocytes than uninjured tissue (Z=−2.809, *P*=0.045) and 3–4 month olds (Z=−3.048, *P*=0.023) (Fig. 4A-B). There were also significantly fewer muscle fibers in the regenerated area than the uninjured tissue for 4–6 months old (Z=−2.17, uncorrected *P* (*P*^#^)=0.039), 6–9 months old (Z=−3.201, *P*^#^=0.001), and ≥22 months old (Z=−2.204, *P*^#^=0.028) (Fig. 4A and D). However, these effects became non-significant after adjusting for multiple tests, except for the 6–9-month-old animals (Table S1). Finally, 4–6-month-old animals regenerated more hairs than 3–4-month-olds (Z=−2.510, *P*^#^=0.012), as did 6–9-month-olds (Z=−2.390, *P*^#^=0.017) and ≥22 months old (Z=2.032, *P*^#^=0.042) (Fig. 4E). However, these effects were non-significant after adjusting for multiple testing (Table S2).

### Adipocytes, muscle fibers, and hairs across spatial regions

The number of adipocytes, muscle fibers, and hairs per 100 µM were also compared spatially from proximal to middle to distal region, and in relation to age. Similar to the spatial analysis for cartilage, three linear mixed models were compared by adding the fixed effects of position, age, and the interaction of position and age for each model. For adipocytes, the model comparison indicated that there was an effect of position [*F*(1, 15)=36.007, *P*<0.001] and age [*F*(3, 10)=4.093, *P*=0.038], with no significant effect of the interaction. Post-hoc analyses indicated there were fewer adipocytes in the distal tissue (t=−5.519, *P*=0.001) (Fig. 4C and F). Muscle fibers, significantly differed spatially [*F*(1, 12)=11.491, *P*=0.006], decreasing proximally to distally (t=−3.390, *P*=0.006) (Fig. 4C and E). Notably, for 6–9-month-old animals, muscle was only present in the proximal tissue. Comparisons on hairs suggested no significant differences across spatial position [*F*(1,13)=0, *P*=0.999] (Fig. 4H).

### Summary of regeneration quality results

**Fig. 5.**
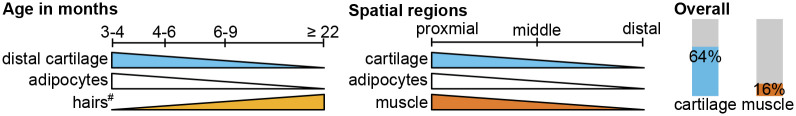
**Summary of quality metrics.** See summary text for details. The # after hairs indicates that this difference was not significant after correcting for multiple testing.

In summary, the quality of tissue regeneration differed depending on age and spatial position within the regenerated tissue (Fig. 5). For age, there was significantly less cartilage in the distal injured region of animals ≥22 months old compared to 3–4 months old (*P*<0.01). Animals ≥22 months old also had significantly less adipocytes than 3–4-month-old (*P*<0.05), and all age groups older than 3–4 months old had more hairs (uncorrected *P*^#^<0.05). Spatially, there was significantly less cartilage (*P*<0.01), adipocytes (*P*<0.001), and muscle fibers (*P*<0.01) in the most distal tissue for all ages. These analyses also determined that compared to uninjured tissue, regardless of age, *Acomys* only regenerated ∼64% of the cartilage with more gaps or foramen in the cartilage and ∼16% of the muscle.

### Minimal evidence for age-related nerve degeneration

**Fig. 6.**
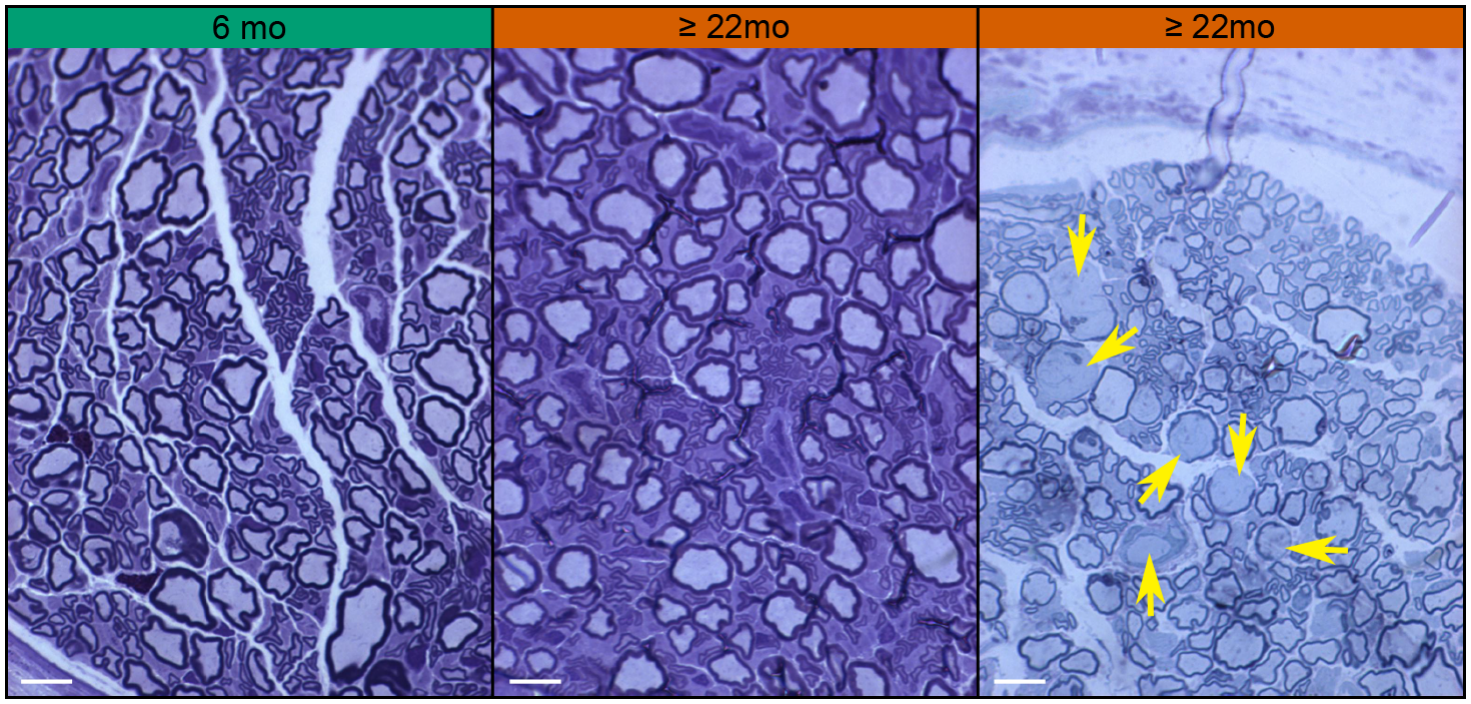
**Auriculotemporal nerves of 6-month-old and ≥22-month-old *Acomys*.** Semithin sections of auriculotemporal nerves stained with Toluidine Blue (for 6 months old, *n*=3 and for ≥22 months old, *n*=4). One sample for the ≥22 month age group had partially and fully demyelinated axons, which are marked with yellow arrows. Scale bars: 10 µM.

In a second study, the auriculotemporal nerves innervating the ear were dissected from 6-month-old and ≥22-month-old *Acomys* and processed for semi-thin sections. We found no stark differences between 6-month-old and ≥22-month-old *Acomys* auriculotemporal nerves (Fig. 6A-B). Suggesting no clear signs of age-related partial nerve degeneration or damage. However, one ≥22-month-old animal had some demyelination (Fig. 6C). Thus, some (*n*=1) older *Acomys* can have partial nerve damage.

## DISCUSSION

The research indicated that *Acomys* older than 6 months old had delayed ear wound regeneration after a 4 mm biopsy compared to 3–4-month-olds, and muscle regeneration in the ear wound can be missed when the most proximal tissue is not measured. Some older animals also had more spatially heterogenous regeneration than younger animals. Specifically, there was less cartilage in the distal region and less adipocytes in ≥22 month-old compared to 3–4 month-old animals, and some evidence for more hairs for all ages older than 6 months compared to 3–4 month-old animals. There was little evidence that older animals had age-related nerve degeneration, which we thought could have accounted for delayed regeneration or inconsistencies in muscle regeneration. Interestingly, all *Acomys* regenerated significantly less cartilage, adipocytes, and muscle compared to uninjured tissue, especially in the distal region. This suggests that *Acomys* have imperfect regeneration of their ear tissue. In the remainder of this section, the findings are compared to other regenerative animals, and follow-up experiments are discussed.

Ignoring the age of the animals will likely contribute to irreproducibility in regeneration research in *Acomys*. Here we demonstrated that after a 4 mm ear biopsy, animals older than 6 months old have more delayed regeneration than younger 3–4-month-old *Acomys*, and that *Acomys* ≥22 months-old regenerate less distal cartilage and fewer adipocytes than 3–4 month olds. Current reports of ear-pinna regeneration in *Acomys* have overlooked the importance of age, limiting studies to animals older than 4–4.5 months old (see Table 1). This can be due to various factors including the lengthy gestation time and small litter size (see Introduction). Nonetheless, this study suggests that age must be considered in the experimental design or statistical plans when studying *Acomys* ear-pinna regeneration. Scientists should either standardize the age of animals within an experiment or treat age as a randomized blocking factor, counter-balancing age across experimental groups (e.g. time point or treatment) (Festing, 2020). Future studies should measure differences in signaling pathways, regenerative pathways, and cellular behavior between young and old mice to better understand these caveats and differences. However, in these studies it will be important to compare the phases or stages of regeneration (e.g. blastema formation, cellular differentiation, and cellular proliferation) rather than just days post injury.

Ignoring the spatial region of the regenerated tissue may also contribute to irreproducibility in *Acomys* regeneration research. Here, we showed that the quality of cartilage, muscle, and adipocyte regeneration declines from the proximal to distal region of the injury. This finding is in concordance with a recent molecular study on the spatial regions during regeneration (i.e. 1 h post injury, 5 dpi, 10 dpi, and 55 dpi), which demonstrated spatial heterogeneity of gene expression of many cell types (van Beijnum et al., 2023). However, the study only included animals that had not yet closed their ear hole. The current study adds to this study, by including animals that closed the ear hole and extensive analysis on the presence of cartilage, adipocytes, muscle fibers, and hair follicles. Together, the studies suggest that regeneration is more robust in the proximal region of the injury, and experiments targeting the increased regeneration of cartilage, adipocytes, and muscles in the proximal region may be fruitful for understanding the mechanisms of ear regeneration.

We thought that the older *Acomys* with delayed regeneration had age-related peripheral nerve degeneration since partial nerve damage is known to lead to delayed regeneration with poor quality or patterning defects (Egar et al., 1982; Maden, 1981; Wells et al., 2021). However, the differences in the auriculotemporal nerves between 6-month-old and ≥22-month-old animals were negligible. Previous studies on mice and rats demonstrate that peripheral nerves in 24-month-old rodents are degenerating, have undulated myelin, and have significantly more thinly myelinated axons (Larsson et al., 2019; Yuan et al., 2018). However, both ages appear to have similar levels of undulated myelin and thinly myelinated axons, and only one older animal had demyelinating axons. Thus, it is unlikely that changes in the peripheral nerves across ages account for the differences we observed in timing and quality.

Given that we found negligible levels of age-related nerve degeneration, several other factors, including androgens and growth hormones, could contribute to delayed regeneration. The 3–4 month-old *Acomys* regenerated faster than the older 6-month-old and ≥22-month-old animals. Coincidentally, *Acomys* becomes sexually mature from 2–4 months old (Dieterlen, 1961; Haughton et al., 2016). Thus, puberty-related hormones like androgens and growth hormones may be linked to faster regeneration in *Acomys*. Indeed, growth hormones significantly increase during puberty (Rose et al., 1991) and can accelerate wound healing (Lal et al., 2000) and regeneration (Sun et al., 2011). Also, estrogen accelerates wound healing in female mice (Ashcroft et al., 1997; Hardman et al., 2008) and regeneration in zebrafish (Xu et al., 2020). However, testosterone is known to inhibit wound healing in male mice (Ashcroft and Mills, 2002). Thus, it would be worthwhile to characterize puberty-related hormones in *Acomys* and test their role during ear-hole regeneration.

A secondary finding of this study was that *Acomys* had imperfect regeneration of the ear-pinna, only regenerating around 64% of the cartilage and 16% of the muscle compared to uninjured tissue. Previously, studies have only reported the time to regenerate and general observations on the types of tissues regenerating in the ear-pinna (Gawriluk et al., 2016; Matias Santos et al., 2016; Seifert et al., 2012). Here, we reported on the quality of their regeneration, which was imperfect. It is possible that some of these imperfections could be resolved with time (i.e. longer days post injury) since the tissue that was analyzed was likely still regenerating when collected. However, similar imperfections were observed 221 days post-injury in two 6-month-old *Acomys* (Fig. S1). Other regenerative animals, like reptiles, have imperfect tissue regeneration. The regenerated tail of a lizard is almost entirely cartilaginous, with only an unsegmented cartilaginous tube rather than the segmented vertebrae (Fisher et al., 2012; Lozito and Tuan, 2015). It also has irregular muscle bundles and no peripheral nerves or dorsal root ganglia beyond the regenerated spinal cord (Fisher et al., 2012). Recent studies in lizards have increased the quality of their tail regeneration with embryonic stem cells (Lozito et al., 2021). Similar experiments could be explored in *Acomys* once cellular and molecular differences are better understood between the spatial regions and ages.

In summary, regeneration timing and quality declined with age, and *Acomys* had imperfect regeneration of their ear-pinna. These differences in timing and quality with age are a significant concern for experiments using *Acomys*. We suggest that studies control for age by either standardizing their studies to a specific age or using a randomized-block design across multiple ages. Studies should also consider the zone (i.e. proximal, middle, or distal) where they take tissue for analysis since regeneration quality declines in the more distal tissue, agreeing with recent reports (van Beijnum et al., 2023). We were surprised by the lack of age-related peripheral nerve regeneration in 6–≥22-month-old *Acomys*. More research on multiple ages and other peripheral nerves will be necessary to corroborate this evidence. Also, it remains unclear what underlying factor leads to delayed regeneration and poor regeneration quality in the distal zone. Nonetheless, we believe this evidence suggests that *Acomys* are an excellent model for studying the role of age on regeneration to develop therapies that maximize regeneration quality in older adult humans receiving regenerative treatments.

## MATERIALS AND METHODS

### Subjects

All spiny mice (*Acomys cahirinus*) were bred from our colony at the University of Florida, and most were part of another experiment on the social dominance behavior of *Acomys* (Varholick et al., 2023). All procedures were approved by the Institutional Animal Care and Use Committee of the University of Florida (protocol codes #201807707 and #202110500). For the study on timing, 25 male and female *Acomys* (15 males and 10 females) were housed in same-sex groups of two or three and ranged in age from 3 to ≥22 months. Each animal within the cage was identified with a micro-tattoo on the distal edge of their ear pinna. When dividing the animals into age categories, seven were in the 3–4-month category, seven were in the 4–6-month category, seven were in the 6–9-month category, and four were in the ≥22-month category. The specific ages were variable due to the low litter-size of *Acomys* and the need to injure the animals in reasonably sized batches. Only four subjects from each age category were analyzed for quality of regeneration. A second study of four 6 month-olds and four ≥22 month-olds (two males and two females for each age group) were used for the age-related never degeneration portion of the study. No *a priori* sample size calculation was conducted, given the study's exploratory nature.

### Ear-punch biopsy and regeneration timing

Animals were anesthetized with 4% (v/v) vaporized isoflurane (Pivetal^®^, Patterson Vet Supply) and provided 5 mg/kg of meloxicam subcutaneously for analgesia. A through-and-through hole was made in the left or right ear pinna using a 4-mm biopsy punch (Robbins Instruments, Chatham, NJ, USA) ∼1 mm distal from the head and centered on the middle of the pinna. Following injury, the animals were lightly anesthetized for several minutes every 5 days until the hole was closed for measurements. Measurements were made using calipers, measuring each ear hole's diameter of the proximal-distal (DPD) and the anterior-posterior (DAP) axes. The ear-hole area was calculated for an ellipse to account for unevenness across the hole (Eqn 1). It was considered closed when no light could be seen through the hole in the ear. Experimenters were blinded to the age of the animals.
(1)

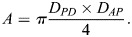


### Regeneration quality

After the ear-hole closed, the entire ear was collected, fixed in 10% neutral buffered formalin, and processed for paraffin sectioning. The regenerated region of each ear was then serially sectioned on a microtome at 10 µM from proximal (nearest the head or base of the ear) to distal (furthest from the head or base of the ear). Every third slide was then stained with Hematoxylin and Eosin (H&E). The stained sections were then used to determine the quality of regeneration. Photographs of one section from each H&E slide were taken at 10× or 20× and stitched together to have a single image of the section. Cartilage quality was determined by measuring the length of the regenerated cartilage relative to the length of the regenerated tissue in QuPath (version 0.4.4) (Bankhead et al., 2017). If there were multiple pieces of cartilage in the section, then the length of each section of cartilage was measured and summed. Adipocytes were also counted manually in QuPath for the same sections, and the number was divided by the length of the regenerated tissue and divided again to get the number of adipocytes per 100 µM. Muscle fibers were counted manually at 40× and were calculated as the number of fibers per 100 µM. Hairs were also counted at 40× using the same method. The same metrics were taken for the uninjured control tissue on 4 mm wide regions of interest. Experimenters were blinded to the age of the animal.

### Age-related nerve damage

This portion of the research aimed to visualize nerve degeneration within *Acomys* auriculotemporal nerves, which are a branch of the trigeminal nerve (i.e. cranial nerve V) and innervate the ear-pinna. Immediately after sacrifice, an incision was made parallel and behind the base of the ear to dissect the auriculotemporal nerve, running from the caudal region of the brain to the ear (Buckley et al., 2012) (Fig. S2). The nerve was fixed in Trump's fixative [Electron Microscopy Sciences (EMS), Hartfield, PA, USA]. All subsequent steps were microwave-assisted and processed with a Pelco BioWave Pro laboratory microwave (Ted Pella, Redding, CA, USA). The nerves were washed with 0.1 M sodium cacodylate buffer containing 2 mM MgCl_2_, 1 mM CaCl_2_, 0.25% NaCl, pH 7.24, post-fixed with buffered 2% osmium tetroxide (OsO_4_), water washed and dehydrated in a graded ethanol series of 25% to 100% in 5% increments followed by 100% anhydrous acetone. Dehydrated samples were infiltrated with Araldite/Embed (EMS, Hatfield, PA, USA) and Z6040 embedding primer (EMS, Hatfield, PA, USA) in increments of 3:1, 1:1, 1:3 anhydrous acetone: Araldite/Embed followed by 100% Araldite/Embed. Semi-thick (500 nm) sections were collected using the Leica Artos ultramicrotome (Leica Microsystems, Deerfield, IL, USA) and the DiATOME Ultra 45° diamond knife (DiATOME, Hatfield, PA, USA). Semi-thick sections were stained with Toluidine Blue and imaged with the Keyence BZ-X8000 (Keyence Corp. of America, Itasca, IL, USA).

### Statistical analyses

All statistical analyses were exploratory, performed in R (version 4.3.1), and used a *P*<0.05 as the critical threshold. The normality and homogeneity of variance in each dataset were examined graphically, and no data transformations were performed. To determine differences in timing, a mixed growth model was performed with the lme4 package (Bates et al., 2014; Mirman, 2016). This model was used instead of a repeated measures ANOVA to account for individual animals housed in the same cage. The experimental unit was a single animal nested within their cage; the day post-injury was treated as a repeated measure, and the age at injury was treated as a continuous variable. To determine differences in quality, a one-way ANOVA (i.e. parametric) or Kruskal–Wallis test (i.e. non-parametric) was used to compare the uninjured tissue with the various ages using base R. The mean values across all slides per individual were used for these analyses. The uninjured tissue and the different ages were categorized as a treatment (i.e. dependent variable) with five categorical levels: uninjured, 3–4 months old, 4–6 months old, 6–9 months old, and ≥22 months old. Post-hoc tests were performed when necessary, using Tukey honest significant differences tests for the ANOVA and Dunn's test for the Kruskal–Wallis using the FSA package (Dunn, 1964; Ogle et al., 2023). These comparisons were corrected for multiple testing using a 95% confidence interval for the Tukey tests and a Hochberg correction for the Dunn's test (Hochberg, 1988). To determine differences in quality from proximal to distal position, the uninjured tissue was removed from the analysis. Age-related differences were compared using a linear mixed model using the lme4 package (Bates et al., 2014). Model comparisons were used to determine the simplest model that accounted for the most variation. First, a mixed model with ordinal position (i.e. proximal, middle, or distal) as a fixed effect and position nested within animal-id as a random effect was run as model zero. The fixed effect of age (i.e. 3–4 months old, 4–6 months old, 6–9 months old, and ≥22 months old) was then added as model one. Then, the interaction of position and age was added as model two. The best model was reported after the model comparison.

## Supplementary Material

10.1242/biolopen.060565_sup1 Supplementary information
