# Correction: Older 6-9-month-old spiny mice (*Acomys cahirinus*) have delayed and spatially heterogenous ear wound regeneration

**DOI:** 10.1242/bio.061912

**Published:** 2025-03-06

**Authors:** Justin A. Varholick, Jazmine Thermolice, Gizelle Godinez, Vanessa Dos Santos, Rishi Kondapaneni, Malcolm Maden

Following the publication of this article *Biol. Open* (2024) **13**, bio060565 (doi:10.1242/bio.060565), a reader pointed out the omission of a relevant reference and ambiguity regarding the age of older mice.

The article PDF and online full-text version have been updated.

The authors apologise for these errors, which do not impact the results and conclusions of the paper.

This reference, Tomasso, A., Koopmans, T., Lijnzaad, P., Bartscherer, K., Seifert, A.W. (2023) An ERK-dependent molecular switch antagonizes fibrosis and promotes regeneration in spiny mice *(Acomys).Sci. Adv.* **9**, (doi:10.1126/sciadv.adf2331) has been included as follows:

**Introduction (corrected):** …but only one of four studies included blood measurements or lactating females. One study showed that ear hole punches closer to the head regenerate faster with higher levels of extracellular signal-regulated kinase (ERK)-mediated signaling compared to distal injuries (Tomasso et al., 2023). But most studies employ more distal injuries to our understanding.

… Thus, muscles, for example, may only regenerate in the proximal region. This may also be linked to higher ERK activity in the proximal region of the injury, since the distal injured region is known to have less sustained ERK activity (Tomasso et al., 2023).

**Discussion (corrected):** This finding is in concordance with recent molecular studies on the spatial regions during regeneration (i.e. 1 h post injury, 5 dpi, 10 dpi, and 55 dpi), which demonstrated spatial heterogeneity in ERK activity and of gene expression of many cell types (Tomasso et al., 2023; van Beijnum et al., 2023).

For clarity, the title, abstract and several sections of the article have been amended to specify the age of older animals.

**Title (corrected):** Older 6-9-month-old spiny mice (*Acomys cahirinus*) have delayed and spatially heterogenous ear wound regeneration

**Title (original):** Older spiny mice (*Acomys cahirinus*) have delayed and spatially heterogenous ear wound regeneration

**Abstract (corrected):** Some report that ≥3-years and 9-week old spiny mice have delayed regeneration compared to 2-month and 3-week old mice, respectively, without investigation on the regenerative capacity of muscle.

… the regeneration of muscle, cartilage and adipocytes was spatially heterogeneous, regardless of age declining in amount from the proximal to distal region of the regenerated tissue.

**Abstract (original):** Some report that older spiny mice have delayed regeneration without investigation on the regenerative capacity of muscle.

… the regeneration of muscle, cartilage and adipocytes was spatially heterogeneous, declining in amount from the proximal to distal region of the regenerated tissue.

**Introduction (corrected):** Two studies suggested that age is a significant factor because older, ≥3-years and 9-week *Acomys* had delayed regeneration compared to younger, 2-month and 3-week *Acomys*, respectively (Brewer et al., 2021; Oyake and Mekada, 2024).

… Here, we investigated the relationship between age, timing, and regeneration quality in the *Acomys* ear in 3–4, 4–6, 6–9, and ≥22 month-olds. We also investigated the presence of age-related partial nerve damage in the auriculotemporal nerve innervating the *Acomys* ear. We measured the time to complete regeneration (i.e. closure of a 4 mm biopsy punch to the ear-pinna) in *Acomys* ranging in age from 3 to 9 months. Another set of *Acomys* ≥22 months (i.e. 22 to 34 months) were only measured for the first 35 days of ear hole closure. After regeneration, the tissue from all ages was fixed and serially sectioned to determine the quality of the regenerated cartilage, adipocytes, muscle fibers, and hairs across …

**Introduction (original):** Two studies suggested that age is a significant factor because older *Acomys* had delayed regeneration compared to younger *Acomys* (Brewer et al., 2021; Oyake and Mekada, 2024).

… Here, we investigated the relationship between age, timing, and regeneration quality in the *Acomys* ear. We also investigated the presence of age-related partial nerve damage in the auriculotemporal nerve innervating the *Acomys* ear. We measured the time to complete regeneration (i.e. closure of a 4 mm biopsy punch to the ear-pinna) in *Acomys* ranging in age from 3 to 34 months. After regeneration, the tissue was fixed and serially sectioned to determine the quality of the regenerated cartilage, adipocytes, muscle fibers, and hairs across …

**Discussion (corrected):** There was little evidence that ≥22-month-old animals had age-related nerve degeneration …

Here we demonstrated that after a 4 mm ear biopsy, 4–6- and 6–9-month-old animals have more delayed regeneration than younger 3–4-month-old Acomys …

Future studies should measure differences in signaling pathways, regenerative pathways, and cellular behavior between 3–4-month old and 6–9-month-old and ≥22-month-old mice to better understand these caveats and differences.

… In summary, our results suggest that regeneration timing and quality declined as animals got older, and *Acomys* had imperfect regeneration of their ear-pinna.

**Discussion (original):** There was little evidence that older animals had age-related nerve degeneration **…**

Here we demonstrated that after a 4 mm ear biopsy, animals older than 6 months old have more delayed regeneration than younger 3–4-month-old *Acomys* …

Future studies should measure differences in signaling pathways, regenerative pathways, and cellular behavior between young and old mice to better understand these caveats and differences.

… In summary, regeneration timing and quality declined with age, and *Acomys* had imperfect regeneration of their ear-pinna.

