# Supplementary material for: Older spiny mice (Acomys cahirinus) have delayed and spatially heterogenous ear wound regeneration
Source: Biol Open. 2024 Oct 10;13(10):bio060565. doi: 10.1242/bio.060565 (PMC11554262; doi:10.1242/bio.060565)
Supplement: Supplementary information [file biolopen-13-060565-s1.pdf]

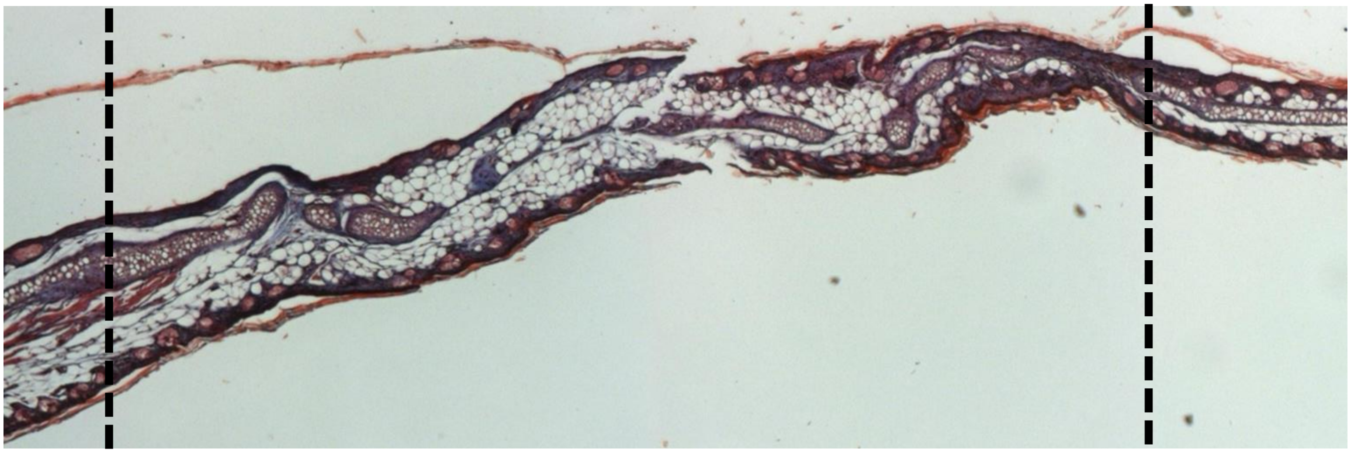

Fig. S1. Ear-pinna 221 days post-injury. At 6mo, two male *Acomys* were injured with a 4mm biopsy punch, and tissue was collected 221 days post-injury. The tissue was stained with Masson's Trichrome stain. Imperfections in the cartilage and the general lack of muscle can be seen in the image. Two vertical lines denote the injury planes. The left side of the image is the proximal tissue, and the right side is the distal tissue.

Biology Open • Supplementary information

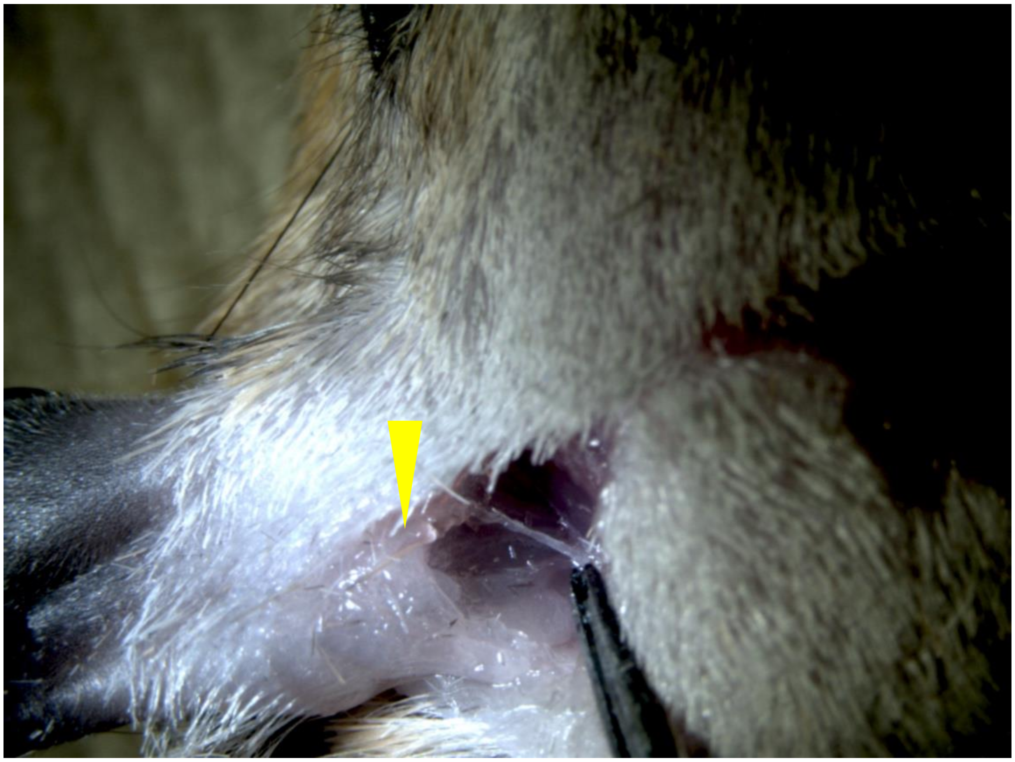

Fig. S2. Auriculotemporal nerve in *Acomys*. This is a photograph identifying the auriculotemporal nerve in an *Acomys* just prior to dissection, after the incision. The nerve is marked with a pointing yellow triangle.

Biology Open • Supplementary information

Table S1. Pairwise comparisons of muscle counts between age groups using Dunn's test, adjusted using Hochberg correction

**Comparison**	**Z**	**p**	**p adjusted**
3-4mo v. Uninj.	-1.82	0.069	0.481
**4-6mo v. Uninj.**	**-2.17**	**0.030**	0.240
**6-9mo v. Uninj.**	**-3.20**	**0.001**	**0.013**
**≥ 22mo v. Uninj.**	**-2.20**	**0.028**	0.248
3-4mo v. 4-6mo	0.35	0.726	1.000
3-4mo v. 6-9mo	1.71	0.086	0.518
3-4mo v. ≥ 22mo	0.52	0.604	1.000
4-6mo v. 6-9mo	1.43	0.153	0.765
4-6mo v. ≥ 22mo	0.194	0.604	1.000
6-9mo v. ≥ 22mo	-1.19	0.233	0.931

Table S2. Pairwise comparisons of hair counts between age groups using Dunn's test, adjusted using Hochberg correction

**Comparison**	**Z**	**p**	**p adjusted**
3-4mo v. Uninj.	-0.837	0.403	1.000
4-6mo v. Uninj.	1.673	0.094	0.660
6-9mo v. Uninj.	1.554	0.120	0.721
≥ 22mo v. Uninj.	1.195	0.232	1.000
**3-4mo v. 4-6mo**	**-2.510**	**0.012**	0.121
**3-4mo v. 6-9mo**	**-2.390**	**0.016**	0.151
**3-4mo v. ≥ 22mo**	**-2.031**	**0.042**	0.337
4-6mo v. 6-9mo	0.120	0.905	0.905
4-6mo v. ≥ 22mo	0.478	0.633	1.000
6-9mo v. ≥ 22mo	0.359	0.720	0.121

Biology Open • Supplementary information
